# Daily diurnal temperature range associated with emergency ambulance calls: a nine-year time-series study

**DOI:** 10.3389/fpubh.2024.1454097

**Published:** 2024-10-03

**Authors:** Chaohui Guo, Keke Cai, Gao Chen, Jin Wang, Jie Zeng, Xiaoqing Huang, Mengling Deng

**Affiliations:** ^1^Department of Clinical Psychology, The Third Hospital of Quzhou, Quzhou, China; ^2^Department of Traditional Medicine, The Affiliated Guangdong Second Provincial General Hospital of Jinan University, Guangzhou, China; ^3^Department of Internet Medical Center, The Affiliated Guangdong Second Provincial General Hospital of Jinan University, Guangzhou, China; ^4^Department of Psychiatry, The Third Hospital of Quzhou, Quzhou, China

**Keywords:** emergency ambulance calls, China, diurnal temperature range, time-series analysis, generalized additive models (GAMs)

## Abstract

**Background:**

Diurnal temperature range (DTR) is associated with the increased risk of morbidity and mortality. However, the relationship between DTR and emergency ambulance calls (EACs), which more accurately and immediately reflect the health impacts of temperature changes, remains underexplored in China.

**Methods:**

We collected daily data on EACs and meteorological factors from 2009 to 2017 in Guangzhou, China. DTR, representing the temperature range within a day, was calculated by subtracting the minimum temperature from the maximum temperature for each day. Generalized additive models were used to estimate the association between DTR and EACs for all-cause, cardiovascular diseases, and respiratory diseases. Additionally, subgroup and sensitivity analyses were conducted in our study.

**Results:**

We found significant associations between daily DTR and EACs. The excess risks (ERs) were 0.47% (95% CI: 0.14, 0.81%) for all-cause EACs, 0.94% (95% CI: 0.46, 1.43%) for cardiovascular-related EACs, and 1.31% (95% CI: 0.76, 1.86%) for respiratory -related EACs at lag01, respectively. Subgroup analyses indicated that these associations were notably stronger among the older, males, and during the warm season. Specifically, there was an increase of 1.16% (95% CI: 0.59, 1.74%) in cardiovascular-related EACs among the older adult, compared to 0.45% (95% CI: −0.21, 1.12%) among those younger than 65 years. Among males, the increase was 1.39% (95% CI: 0.79, 1.99%), compared to 0.13% (95% CI: −0.53, 0.79%) among females. During the warm season, the increase was 1.53% (95% CI: 0.74, 2.34%), compared to 0.75% (95% CI: 0.14, 1.37%) during the cold season.

**Conclusion:**

DTR might increase the risk of daily all-cause, cardiovascular-related, and respiratory-related EACs in Guangzhou, China. The associations were particularly strong among older adults, males, and during the warm season. Implementing public health policies is essential to mitigate the adverse health effects of DTR.

## Introduction

Climate change, driven primarily by human activities, is exerting growing adverse effects on human health (1). Numerous studies over the past decade have highlighted these impacts, suggesting an increased burden of disease and mortality associated with climate change (2, 3). Elevated temperatures can cause heat stress, dehydration, and cardiovascular issues (4). Conversely, cold spells have been closely associated with higher mortality rates (5). These contrasting effects illustrate the intricate and diverse ways in which climate change influences human health.

The diurnal temperature range (DTR), quantified as the variance between daily maximum and minimum temperatures over 24-h periods, serves as a novel metric of climate change and a significant risk factor for human health (6, 7). For example, one study in a Mediterranean region reported that the incidence rate ratio was 1.03 (95% CI: 1.01, 1.06) for extreme high DTR (8). Aghababaeian et al. (9) found that the DTR was associated with the hospital admission on respiratory disease and cardiovascular disease in Dezful, Iran. Besides, one time-series study in Bangkok observed that short-term DTR exposure have impact on elevated risk of hospital admissions due to cardiovascular disease (10).

Previous studies have primarily examined on the relationship between DTR and hospital admissions, morbidity, and mortality (8, 11–13). However, emergency ambulance calls (EACs) might be more sensitive to the acute human health effects, making them a more suitable indicator for reflecting the immediate impacts of short-term exposure to DTR (14). For instance, a multi-city study in Japan indicated that EACs might provide a more appropriate endpoint for observing the acute health effects of climate change (15). Nevertheless, few studies have estimated the association between DTR and EACs for all-cause, cardiovascular, and respiratory diseases.

We thus conducted this time-series study aiming to assess the association between DTR and EACs in Guangzhou, China. We hypothesize that DTR can elevate the EACs.

## Methods

### Study area

Guangzhou, nestled in Southern China, boasts a subtropical humid-monsoon climate, characterized by an annual average temperature of 22°C, rainfall of 1800 millimeters, and a relative humidity of 80%. Guangzhou, as the capital city of Guangdong Province, had a population of 18.6 million in 2020. The large number of people provided sufficient statistical power of observation, resulting in higher quality health outcome data (16).

### Outcome data

The data about all-cause, cardiovascular-related, and respiratory-related EACs were obtained from the Guangzhou Emergency Center spanning the period from January 2009 to December 2017. As the primary emergency dispatch agency in Guangzhou, this center coordinates approximately 200 ambulances and ensures emergency responses within half an hour after receiving an emergency call, serving over 10,000,000 residents, regardless of the time of day (17).

After each emergency call, trained medical personnel completed a standardized data entry form, including demographic information, clinical diagnoses, and main symptoms. The disease outcomes were diagnosed by physicians according to patients’ symptoms, medical inquiries, and examinations, adhering to standardized procedures with rigorous quality assurance and control protocols. Significantly, EACs resulting from suicides, traumatic accidents, and events related to pregnancy or childbirth were excluded from our analysis. Experienced emergency physicians diagnosed cardiovascular and respiratory events based on observed symptoms and signs, maintaining a low rate of misclassification (18).

### Meteorological factor

Meteorological factors, including daily maximum, mean, and minimum temperatures, as well as relative humidity and wind speed, were obtained from the National Weather Data Sharing System. Data from the Guangzhou weather station was utilized to represent the daily exposure of the general population. Following definitions from prior studies (19, 20), the DTR was calculated by subtracting the minimum temperature from the maximum temperature on the same day.

### Statistical models

The short-term association between DTR and daily EACs due to all-cause, cardiovascular diseases, and respiratory diseases was assessed using generalized additive models (GAM) (21). We controlled for potential confounders such as temporal trends, day of the week (DOW), public holidays (PH), daily mean temperature, relative humidity, and wind speed. Temporal trends were adjusted using natural cubic splines with 6 degrees of freedom (df) per year, while daily mean temperature and relative humidity were controlled with 3 df each (22). The model was defined as below:


$$\begin{array}{l} \log\left[E\left(Y_{t}\right)\right]=\beta *DTR+s\left(\mathrm{t,df}=6/year\right)\\ +s\left(Temp03\mathrm{,df}=3\right)+as.factor\left(DOW\right)\\ +s\left(\mathrm{RH,df}=3\right)+s\left(\mathrm{WD,df}=3\right)+as.factor\left(PH\right) \end{array}$$

In the core model, *E*(*Y*_t_) represents the expected number of EACs on day t. The coefficient of the DTR is denoted by 𝛽. The functions () is a smoothing function, and *t* accounts for long-term and seasonal trends. Temp03 is the moving average of the temperature over the previous 3 days. DOW indicates the day of the week. RH and WD represent the relative humidity and wind speed, respectively. PH is a binary variable indicating the public holiday.

We assessed the possible adverse effects of DTR using different lag structures. Single-lag day models considered lag effects from the same day (lag0) up to 5 days lag (lag5). Multi-day lag models evaluated accumulated effects using moving averages for the current day and the previous 1–5 days (lag01, lag02, lag03, lag04, and lag05).

### Subgroup analyses

To check whether the effects of DTR on EACs varied by age group (age < 65 vs. age ≥ 65), sex (male vs. female), and season (cold vs. warm), we conducted analyses stratified by these strata. Based on previous studies (17, 21), the warm seasons defined as April to September, and the cold seasons as the period from October to March of the following year. We determined groups differences by calculating the 95% confidence intervals (CI) as described below:


$$D_{1}-D_{2}\pm 1.96\sqrt{{\left({\mathrm{SE}}_{1}\right)}^{2}+{\left({\mathrm{SE}}_{2}\right)}^{2}}$$

where D_1_ and D_2_ are the estimates for the two strata, and SE_1_ and SE_2_ represent their corresponding standard errors (18).

We performed two sensitivity analyses to ensure the robustness of our main findings. First, we altered the df for temporal trends and meteorological factors, ranging from 5 to 8 and from 4 to 6, respectively. To address the interactions between ambient air pollutants and temperature on EACs (23), we have further adjusted air pollutants, including PM_2.5_, PM_10_, O_3_, NO_2_, and SO_2_, in our main models.

We reported results as excess relative risk (ER) with 95% CI, the ER was calculated as (relative risk [RR] − 1) * 100%. Statistical analyses were performed using R version 4.3.1. Statistical significance was defined as a *p*-value less than 0.05.

## Results

Table 1 displays the means, standard deviations (SDs), percentiles of daily EAC counts, and meteorological factors in our study. During the study period, a total of 914,304 EACs from all causes were recorded, including 85,484 EACs from cardiovascular diseases and 61,034 EACs from respiratory diseases. The daily mean counts of EACs due to all causes, cardiovascular diseases, and respiratory diseases were 320.8, 30.0, and 21.4, respectively. The mean DTR during the study period was 7.7°C (SD: 3.0), with a median DTR of 7.7°C (interquartile range: 5.6–9.6°C). The daily averages for temperature, relative humidity, and wind speed were 22.6°C, 78.1%, and 2.1 m/s, respectively.

**Table 1:** Summary statistics of daily emergency ambulance calls and meteorological variables in Guangzhou, China, from 2009 to 2017.

	Mean	SD	Percentile				
			Min	25th	50th	75th	Max
No. of daily EACs							
All-cause	320.8	80.5	2.0	283.0	330.0	372.0	553.0
Cardiovascular	30.0	10.3	0.0	24.0	30.0	37.0	68.0
Respiratory	21.4	8.0	0.0	16.0	21.0	26.0	52.0
Meteorological variables							
Temperature, °C	22.6	6.2	3.4	18.2	24.4	27.7	32.3
DTR, °C	7.7	3.0	1.0	5.6	7.7	9.6	18.6
Relative humidity, %	78.1	11.6	27.0	72.0	80.0	86.0	100.0
Wind Speed, m/s	2.1	1.0	0.1	1.4	1.9	2.6	8.1

**Figure 1:**
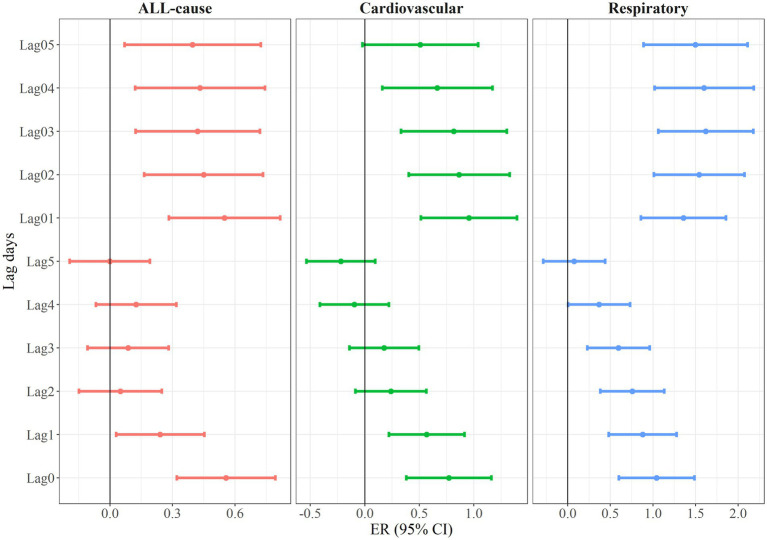
Excess risk (and 95% confidence interval) of emergency ambulance calls per 1°C increment in diurnal temperature range at different lag days.

Figure 1 illustrates the estimates and 95% CI of EACs due to all-cause, cardiovascular diseases, and respiratory diseases for each 1°C increment in DTR. This is presented across different lag days (lag0 to lag5) and moving averages (lag01 to lag05). In general, we observed a largest and robust effect on lag01, so in the subsequent analyses, we mainly reported the effects of lag01. We observed significant effects of DTR on all-cause, cardiovascular-related, and respiratory-related EACs in Guangzhou. Specifically, for each 1°C increase in DTR at lag01, there was a 0.47% (95% CI: 0.14, 0.81%) increase in all-cause EACs, a 0.94% (95% CI: 0.46, 1.43%) increase in cardiovascular-related EACs, and a 1.31% (95% CI: 0.76, 1.86%) increase in respiratory-related EACs. Similar lagged effect patterns were observed for DTR with all-cause and cause-specific EACs. In the single-day lag structures, nearly all DTR-EAC associations decreased from lag0 to lag5. In the moving average lags pattern, the effects decreased gradually from lag01 to lag05, with the largest effects observed at lag01.

**Figure 2:**
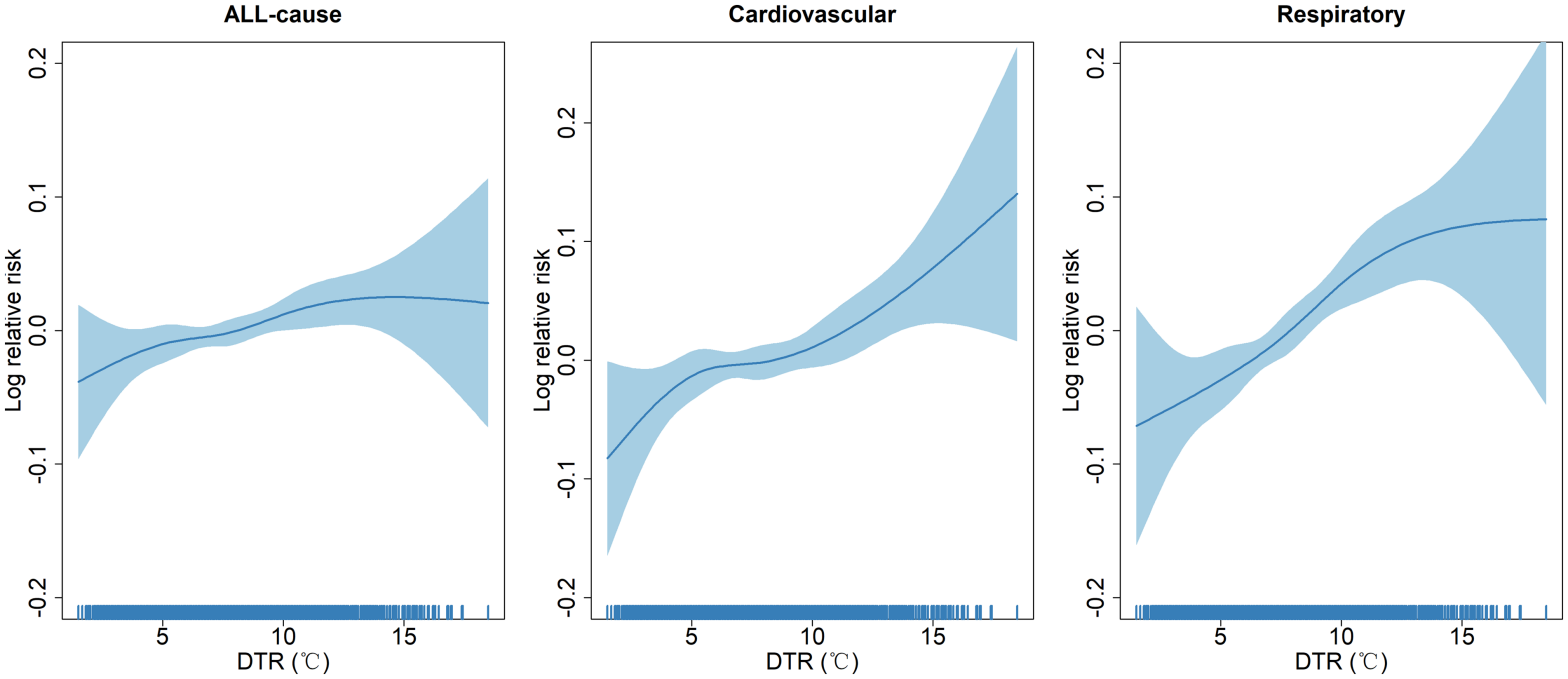
Concentration-response curves showing the non-linear association between diurnal temperature range and log relative risks of emergency ambulance calls.

To further investigate potential non-linear associations between DTR and the risk of EACs from all-cause, cardiovascular diseases, and respiratory diseases, we employed non-linear spline models to estimate the dose–response curves for DTR-EAC associations. Figure 2 reveals nearly linear relationships between DTR and the log-relative risks of EACs due to all causes, cardiovascular diseases, and respiratory diseases.

We further examined the associations between DTR and the risk of EACs due to all causes, cardiovascular diseases, and respiratory diseases, stratified by age, sex, and seasons to estimate potential effect modification. Table 2 indicates significant effect modification by age, sex, and seasons. The effects of DTR on EACs due to all causes, cardiovascular diseases, and respiratory diseases were significantly higher among males, the older adult, and during warm seasons. Specifically, we observed an increase of 1.16% (95% CI: 0.59, 1.74%) in cardiovascular-related EACs for each 1°C increase in DTR among the older adult, compared to 0.45% (95% CI: −0.21, 1.12%) among residents younger than 65 years. Additionally, we found an increase of 1.39% (95% CI: 0.79, 1.99%) in cardiovascular-related EACs among males, compared to 0.13% (95% CI: −0.53, 0.79%) among females. Moreover, there was a 1.53% (95% CI: 0.74, 2.34%) increase in cardiovascular-related EACs during warm seasons, compared to 0.75% (95% CI: 0.14, 1.37%) during cold seasons.

**Table 2:** Excess risk and 95% confidence intervals of emergency ambulance calls due to all-cause, cardiovascular diseases, and respiratory diseases for each 1°C increment in DTR stratified by age group, sex, and season.

Stratum	All-cause		Cardiovascular		Respiratory	
	ER (95% CI)	*P*_interaction_	ER (95% CI)	*P*_interaction_	ER (95% CI)	*P*_interaction_
Age		0.25		**<0.01**		**<0.01**
<65	0.29 (−0.10, 0.68)		**0.45 (−0.21, 1.12)**		**0.37 (−0.45, 1.20)**	
≥65	0.68 (0.28, 1.07)		**1.16 (0.59, 1.74)**		**1.66 (1.02, 2.30)**	
Sex		0.45		**<0.01**		0.76
Male	0.54 (0.17, 0.92)		**1.39 (0.79, 1.99)**		1.36 (0.68, 2.04)	
Female	0.32 (−0.07, 0.71)		**0.13 (−0.53, 0.79)**		1.28 (0.50, 2.07)	
Season		0.88		**<0.01**		0.68
Warm	0.50 (0.22, 0.78)		**1.53 (0.74, 2.34)**		1.35 (0.69, 2.01)	
Cold	0.50 (0.04, 0.96)		**0.75 (0.14, 1.37)**		1.24 (0.52, 1.98)	

The bold texts represent the statistically significant differences (*p* < 0.05).

Our sensitivity analyses, which altered the dfs for temporal trend and meteorological factors, yielded minimal alterations to the effect estimations (Supplementary Table S1), indicating the robustness of our main results. Secondly, we reconstructed our models by adjusting air pollutants and the results agreed with the main findings (Supplementary Table S2).

## Discussion

This time-series study, conducted over 9 years in Guangzhou, China, included 914,304 emergency ambulance calls (EACs). We found that a higher DTR was significantly associated with increased risks of EACs for all-cause, cardiovascular diseases, and respiratory diseases. Subsequent dose–response analyses revealed nearly linear relationships between DTR and all-cause, cardiovascular-related, and respiratory-related EACs. Additionally, we identified notable association modifications by age, sex, and season, with elevated ERs observed among the older adult, male individuals, and during warm seasons compared to those under 65 years of age, female individuals, and cold seasons, respectively. These findings persisted when alternative dfs were applied to temporal trend and meteorological variables.

Our findings align with multiple studies investigating the associations of DTR and human health (24–26). For example, a time-series study in Hong Kong, China has reported that a 1.70% (95% CI: 0.30, 3.10%) increase in daily cardiovascular mortality each 1°C increment in DTR at lag03 (24). Another study across several cities found that increased DTR at a lag of 6 days was associated with a higher risk of hospitalization for chronic respiratory diseases (RR = 1.09, 95% CI: 1.08, 1.11) (25). However, these epidemiologic studies mainly focused on mortality or hospital admissions. Few have examined on EACs, which are more suitable indicator for assessing the immediate health effects of short-term DTR exposure. Our study partly addresses the knowledge gap between DTR and EACs and reconfirms that positive associations exist in accordance with previous studies.

Several underlying biological mechanisms may illustrate the associations between DTR and EACs. First, short-term fluctuations in DTR can induce oxidative stress and inflammation, which are known to exacerbate both cardiovascular and respiratory events (27–30). Second, the cardiovascular system must constantly adjust to these temperature changes, which can increase heart rate and blood pressure, potentially triggering cardiovascular events in vulnerable populations (31). Third, sudden temperature shifts can impair respiratory function by causing bronchoconstriction and increasing the susceptibility to respiratory infections, thus heightening the risk of respiratory diseases (6).

We found that DTR had a larger effect on cardiovascular-related EACs among the older residents compared to younger residents, which consistent with previous studies (19, 26). For instance, Amoatey et al. (26) observed a higher impact of DTR among the older population in Victoria state of Australia. It was possible that older individuals have a reduced ability to regulate their core body temperature in response to fluctuating temperatures. Additionally, the higher prevalence of chronic diseases in this age group means that rapid daily temperature changes can place extra stress on their cardiovascular systems, potentially triggering cardiovascular events (32).

We observed that male residents were vulnerable to DTR, which has been widely reported in previous studies (33, 34). For example, a time-series study conducted in China, showed that the greatest effect values were observed in males (ER = 1.35, 95% CI: 0.33, 2.39%) at lag06, compared to females (0.86, 95% CI: 0.24, 1.49%) at lag 01 (33). One possible explanation is the difference in thermoregulation between different gender. Males typically have a higher metabolic rate, which can lead to greater heat production and increased susceptibility to temperature changes (35). Additionally, hormonal differences, such as higher testosterone levels in males, might affect the cardiovascular system, making it more reactive to temperature fluctuations (36). Furthermore, studies suggest that males may have less subcutaneous fat than female, reducing their insulation against temperature extremes and increasing their vulnerability to climate changes (37).

Consistent with previous study (11, 12, 38), our findings indicate that DTR had a more detrimental health effect during warm seasons. Several underlying mechanisms may explain these observed associations. A plausible explanation involves the influence of temperature on the body’s thermoregulatory capabilities. High temperature can disrupt sweat evaporation, resulting in diminished cooling efficiency and heightened thermal stress on the body. This heightened stress can raise heart rate and blood pressure, thereby increasing the risk of cardiovascular events (39, 40). Additionally, higher temperatures may enhance oxidative stress in the body, leading to cellular damage and inflammation, which are risk factors for cardiovascular diseases (41, 42).

There are several limitations to this study. First, as a time-series analysis of daily EACs, the results are susceptible to potential ecological fallacy bias (43). Second, some important covariates, including socioeconomic status, living environment, and lifestyle, were unavailable in our study. Third, our study was performed in a single city in China due to data accessibility, limiting the generalizability of the findings.

## Conclusion

Our study indicates that DTR could elevate the risk of the EACs due to all-cause, cardiovascular diseases, and respiratory diseases. The impact of DTR may be pronounced among the older adult individuals, male residents, and during warm seasons. It underscores the necessity for pertinent public health policies, encompassing educational initiatives and health promotion efforts, to alleviate the adverse health repercussions result from higher DTR.

## Data Availability

The data analyzed in this study is subject to the following licenses/restrictions: please contact author for data requests. Requests to access these datasets should be directed to huangxqgd2h@163.com.
