# Supplementary material for: Daily diurnal temperature range associated with emergency ambulance calls: a nine-year time-series study
Source: Front Public Health. 2024 Oct 3;12:1454097. doi: 10.3389/fpubh.2024.1454097 (PMC11484036; doi:10.3389/fpubh.2024.1454097)
Supplement: Supplementary file 1 [file Table_1.DOCX]

**Table S1**. Associations between DTR and risks of EACs using different degrees of freedom for spline effects of meteorological factors at lag01.

| Models | All-cause | Cardiovascular | Respiratory |
| --- | --- | --- | --- |
| df=5 for temporal trends | 0.51 (0.25, 0.78) | 0.95 (0.51, 1.40) | 1.35 (0.86, 1.85) |
| df=7 for temporal trends | 0.57 (0.33, 0.82) | 1.00 (0.57, 1.44) | 1.37 (0.87, 1.87) |
| df=8 for temporal trends | 0.44 (0.20, 0.69) | 0.84 (0.41, 1.28) | 1.22 (0.73, 1.72) |
| df=4 for temperature | 0.51 (0.24, 0.78) | 0.94 (0.50, 1.39) | 1.36 (0.86, 1.86) |
| df=5 for temperature | 0.51 (0.25, 0.78) | 0.95 (0.50, 1.40) | 1.35 (0.86, 1.85) |
| df=6 for temperature | 0.55 (0.28, 0.82) | 0.95 (0.51, 1.40) | 1.36 (0.86, 1.86) |
| df=4 for relative humidity | 0.55 (0.28, 0.82) | 0.94 (0.50, 1.38) | 1.36 (0.86, 1.86) |
| df=5 for relative humidity | 0.55 (0.28, 0.82) | 0.93 (0.50, 1.37) | 1.36 (0.86, 1.86) |
| df=6 for relative humidity | 0.55 (0.28, 0.82) | 0.93 (0.50, 1.37) | 1.36 (0.86, 1.86) |
| df=4 for wind speed | 0.55 (0.28, 0.82) | 0.95 (0.51, 1.40) | 1.36 (0.86, 1.86) |
| df=5 for wind speed | 0.55 (0.28, 0.82) | 0.95 (0.51, 1.40) | 1.36 (0.86, 1.86) |
| df=6 for wind speed | 0.55 (0.28, 0.82) | 0.95 (0.51, 1.40) | 1.36 (0.86, 1.86) |

DTR: diurnal temperature range.

**Table S2**. Associations between DTR and risks of EACs after adjusting for air pollutants.

| Models | All-cause | Cardiovascular | Respiratory |
| --- | --- | --- | --- |
| Adjusting for PM_2.5_ | 0.51 (0.20, 0.84) | 1.24 (0.45, 2.04) | 1.30 (0.40, 2.22) |
| Adjusting for PM_10_ | 0.38 (0.06, 0.71) | 1.12 (0.30, 1.94) | 1.22 (0.29, 2.17) |
| Adjusting for NO_2_ | 0.18 (-0.50, 0.86) | 0.91 (0.09, 1.73) | 1.12 (0.18, 2.07) |
| Adjusting for SO_2_ | 0.73 (0.07, 1.39) | 1.42 (0.62, 2.22) | 1.65 (0.74, 2.57) |
| Adjusting for O_3_ | 0.82 (0.15, 1.49) | 1.57 (0.77, 2.38) | 1.80 (0.88, 2.73) |
